# CNTs/CNPs/PVA–Borax Conductive Self-Healing Hydrogel for Wearable Sensors

**DOI:** 10.3390/gels11080572

**Published:** 2025-07-23

**Authors:** Chengcheng Peng, Ziyan Shu, Xinjiang Zhang, Cailiu Yin

**Affiliations:** Guangxi Colleges and Universities Key Laboratory of Environmental-Friendly Materials and New Technology for Carbon Neutralization, Guangxi Key Laboratory of Advanced Structural Materials and Carbon Neutralization, School of Materials and Environment, Guangxi Minzu University, Nanning 530105, China; 18239454619@163.com (C.P.); 15811884977@163.com (Z.S.)

**Keywords:** hydrogel, strain sensing, self-healing

## Abstract

The development of multifunctional conductive hydrogels with rapid self-healing capabilities and powerful sensing functions is crucial for advancing wearable electronics. This study designed and prepared a polyvinyl alcohol (PVA)–borax hydrogel incorporating carbon nanotubes (CNTs) and biomass carbon nanospheres (CNPs) as dual-carbon fillers. This hydrogel exhibits excellent conductivity, mechanical flexibility, and self-recovery properties. Serving as a highly sensitive piezoresistive sensor, it efficiently converts mechanical stimuli into reliable electrical signals. Sensing tests demonstrate that the CNT/CNP/PVA–borax hydrogel sensor possesses an extremely fast response time (88 ms) and rapid recovery time (88 ms), enabling the detection of subtle and rapid human motions. Furthermore, the hydrogel sensor also exhibits outstanding cyclic stability, maintaining stable signal output throughout continuous loading–unloading cycles exceeding 3200 repetitions. The hydrogel sensor’s characteristics, including rapid self-healing, fast-sensing response/recovery, and high fatigue resistance, make the CNT/CNP/PVA–borax conductive hydrogel an ideal choice for multifunctional wearable sensors. It successfully monitored various human motions. This study provides a promising strategy for high-performance self-healing sensing devices, suitable for next-generation wearable health monitoring and human–machine interaction systems.

## 1. Introduction

For wearable electronic devices, flexible pressure sensors offer significant advantages due to their simple structure, high sensitivity, and low power consumption, making them highly promising for applications in health monitoring [1], human–machine interaction [2], and soft robotics [3]. However, traditional rigid sensors made from conductive materials often face challenges in achieving precise and real-time signal detection due to their limited flexibility, stretchability, and recoverability [4]. Additionally, these traditional sensors frequently fail due to fatigue wear and perform poorly in diverse detection scenarios. Flexible sensors made from polyvinyl alcohol (PVA)-borax hydrogels, which exhibit excellent flexibility, self-healing properties, and biocompatibility, have been widely adopted as human motion monitoring sensors [5,6,7]. Many researchers have attempted to combine conductive materials with flexible substrates to prepare stretchable, compressible, and bendable flexible sensors [8,9,10,11,12,13,14,15]. However, such sensors generally suffer from poor interface adhesion and poor skin contact, leading to unstable signal transmission and poor user experience [16,17,18]. However, the practical application of pure PVA–borax hydrogels is limited by insufficient conductivity and mechanical strength, severely restricting their use in high-sensitivity sensors, especially in wearable sensors. Therefore, there is an urgent need to develop efficient functionalization enhancement strategies for such hydrogels to overcome performance bottlenecks.

The self-healing properties of PVA–borax hydrogels stem from dynamic covalent diol–borax bonds and supramolecular interactions, which enable reversible reorganization of the network after damage. Recent research has primarily focused on enhancing these networks through the integration of nanomaterials. For example, by arranging polymer chains and nanoparticles to dissipate stress, high tensile strength [19] has been achieved. Despite some progress, improving the conductivity of PVA–borax hydrogels and optimizing their sensing performance remains a significant challenge. Traditional conductive additives such as salts or polymers are prone to leaching during swelling or healing stages, leading to signal drift [20]. Integrating conductive materials such as carbon nanomaterials (e.g., graphene [21], carbon nanotubes [22], reduced graphene oxide [23], metallic nanoparticles [24], and nanowires [25]) within the hydrogel to form layered, interpenetrating networks. Conductive material-modified PVA–borax hydrogels have become a key pathway to breaking through the performance bottleneck of sensors. The synergistic effects of multi-component conductive fillers are also widely used to enhance the overall performance of hydrogel sensors. Research indicates that combinations of conductive materials with different dimensions can construct more efficient permeable networks, significantly enhancing charge transport capabilities. For example, the composite system of carbon nanotubes (CNTs) and graphene, where one-dimensional CNTs bridge two-dimensional graphene layers, reduces contact resistance and achieves high conductivity at low filler content [26]. Additionally, the synergistic effect of MXene and conductive polymers has been demonstrated to simultaneously optimize mechanical toughness and electrochemical activity [27]. The composite of lignin nanoparticles and cellulose nanofibers enhances the viscoelasticity and thermal stability of hydrogels, exhibiting a porous network structure and excellent recovery behavior [28]. These studies indicate that by rationally designing the geometric morphology of fillers and multi-component synergies, it is possible to achieve simultaneous gains in conductivity, mechanical resilience, and functionality, providing important insights for the development of high-performance self-healing conductive hydrogels.

One of the most notable characteristics of CNTs is their extremely high aspect ratio (typically exceeding 1000) and exceptional intrinsic conductivity. This property confers a significant advantage to CNTs as conductive fillers, enabling them to form interconnected three-dimensional conductive networks in PVA matrices at extremely low addition levels. In addition to serving as conductive pathways, CNTs also function as highly efficient nanoscale reinforcing agents, with inherent high strength and modulus far exceeding those of the PVA matrix. When uniformly dispersed in the PVA matrix, CNTs effectively bear and transmit stress. Nanoscale reinforcing agents enhance the matrix’s crosslink density, mechanical strength, and toughness through strong hydrogen bonding and π-π stacking interactions with PVA chains [29]. Additionally, carbon nanospheres (CNPs) possess a unique zero-dimensional structure, and their high specific surface area and tunable surface chemical properties have garnered significant attention as conductive fillers in hydrogels. Chen et al. [19] prepared lignin-based nanospheres using a soft template and sol–gel hydrothermal method in an alkaline aqueous medium and combined them with PVA to fabricate a flexible pressure sensor, achieving a threefold increase in material tensile strength from 96.8 kPa to 303.1 kPa. By introducing biomass carbon nanospheres as conductive media into the fur-like structure on the surface of polydimethylsiloxane, a high-sensitivity sensor (135.2 kPa^−1^) with a pressure range of 0–1 kPa was obtained [30]. However, zero-dimensional CNPs lack long-range connectivity, and current research still faces issues of insufficient conductive network efficiency. Additionally, high CNP content can cause stress concentration, leading to a decrease in fracture elongation. In the field of functionalizing flexible sensors with zero-dimensional and one-dimensional carbon materials, current research primarily focuses on single CNTs or CNPs functionalizing modified PVA-based hydrogel systems, with relatively few studies exploring the synergistic effects of mixing zero-dimensional/one-dimensional nanocarbon materials.

In recent studies, a variety of one-dimensional (1D) or zero-dimensional (0D) conductive fillers [31] have been introduced into polymer matrices, leading to a better overall performance of flexible sensors. However, single filler systems (e.g., carbon nanotubes [32]) often face limitations such as poor dispersion or insufficient conductive networks (e.g., carbon nanospheres [33]). In this case, 0DCNPs from waste cuttlefish ink act as point fillers, while 1DCNTs act as line fillers. The CNPs effectively fill the gaps between the CNTs to form a more continuous and robust conductive network [34]. The synergistic point–wire architecture not only facilitates the formation of conductive pathways for the sensor’s conductive fillers but also enhances its environmental sustainability and reproducibility.

This study proposes a point–wire synergistic enhancement strategy combining CNTs and CNPs, where one-dimensional CNTs form a conductive network throughout the main structure, while zero-dimensional CNPs act as “conductive bridges” to enhance electron transport between CNTs. The aim is to develop a high-performance CNT/CNP synergistic reinforcement PVA–borax dual-network hydrogel and systematically investigate the hydrogel’s piezoresistive response and mechanical properties (strength, toughness, self-healing ability). The piezoresistive sensors developed using this material have a high sensitivity (10.06 kPa^−1^), a wide detection range (0–28 kPa), fast response/recovery time (88 ms/88 ms), and cycling stability (3200 cycles), successfully applied in scenarios such as human joint motion monitoring and touch recognition. This work not only provides new insights for designing multifunctional hydrogel sensors but also deepens our understanding of the synergistic enhancement mechanisms of diverse nanofillers, thereby advancing the practical application of flexible electronic devices.

## 2. Results and Discussion

### 2.1. Material Characterization

#### 2.1.1. FT-IR Analysis of CNT/CNP/PVA–Borax Mixture

**Figure 1:**
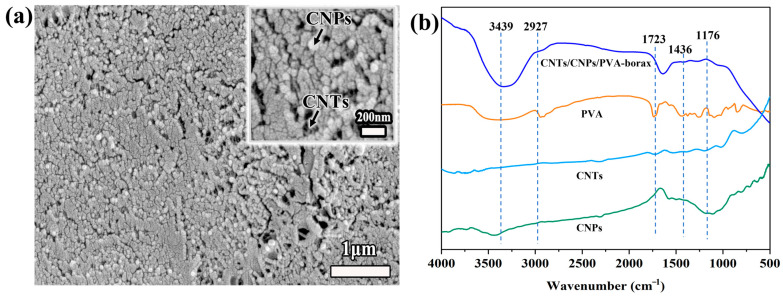
(**a**) SEM image and (**b**) FTIR spectrum of the CNT/CNP/PVA–borax hydrogel.

As shown in Figure 1a, the micro-morphology of the CNT/CNP/PVA–borax hydrogels were observed by SEM, showing a dense porous morphology. The CNTs and CNPs were uniformly distributed on the surface. The inset provides a magnified view to enable further observation of CNTs and CNPs well dispersed in the hydrogel matrix. The carbon nanotubes showed an elongated fibrous structure, while the carbon nanoparticles showed spherical particles. Figure 1b shows the FT-IR spectrum of the CNT/CNP/PVA–borax mixture. Broad and strong absorption peaks were observed at 3438 and 2906 cm^−1^, respectively, attributed to the stretching vibrations of the –OH and C–H groups in PVA. This indicates the presence of a small amount of oxidized functional groups in CNPs and CNTs. Additionally, the absorption peak at 1334 cm^−1^ corresponds to the B–O–C stretching vibration of the borax, indicating that a cross-linking reaction has occurred between the borax and PVA, forming a dynamic covalent structure.

#### 2.1.2. Mechanical Properties of CNT/CNP/PVA–Borax Hydrogel

**Figure 2:**
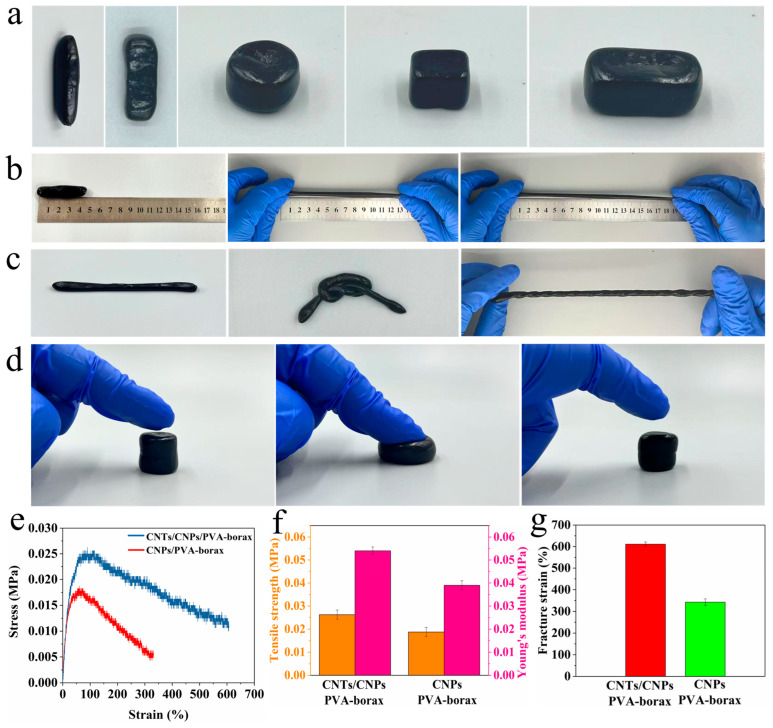
Mechanical properties of CNT/CNP/PVA–borax hydrogel: (**a**) shape adaptability, (**b**) different degrees of tensile deformation, (**c**) knotting and twisting under tensile deformation, (**d**) compression and recovery. (**e**) stress–strain curve, (**f**) tensile strength and modulus of elasticity, and (**g**) fracture strain.

The mechanical properties of hydrogels significantly influence their application scope, making excellent mechanical properties crucial. The prepared CNT/CNP/PVA–borax hydrogel exhibits outstanding shape adaptability, stretchability, twistability, and compressibility. In Figure 2a, the CNT/CNP/PVA–borax hydrogel was shaped into irregular, cylindrical, cubic, and rectangular forms, demonstrating its ability to be reshaped into various shapes. In Figure 2b, the prepared hydrogel can withstand significant stretching, with notable elongation indicating high extensibility. The ability to withstand multiple knotting and twisting operations highlights its excellent flexibility and resistance to shear failure (Figure 2c). Additionally, the CNT/CNP/PVA–borax hydrogel exhibits outstanding shape recovery performance. When finger pressure is applied to the cylindrical hydrogel, it is compressed into a thin sheet but does not rupture. Upon removal of the pressure, it rapidly recovers to nearly its original shape and height (Figure 2d). After removing the applied stress and undergoing a period of rest, the hydrogel can spontaneously and nearly completely recover its original shape and dimensions due to the dynamic borax ester bonds in the PVA–borax network. These dynamic bonds reversibly break and reform, allowing polymer chains to slide and dissipate energy during deformation [35]. After stress release, the dynamic bonds rapidly reform, driving the network back to its thermodynamically favorable equilibrium configuration and restoring its macroscopic original shape. This macroscopic behavior is crucial for practical wearable sensing applications. To investigate the tensile properties of hydrogels, comparative tests were conducted on different samples, as shown in Figure 2e–g. The CNT/CNP/PVA–borax hydrogel exhibited a tensile strength of 0.026 MPa, a Young’s modulus of 0.054 MPa, and an elongation at break of 611%. In contrast, the CNP/PVA–borax hydrogel without CNTs showed a tensile strength of 0.018 MPa, a Young’s modulus of 0.039 MPa, and a fracture elongation of 342%. These results demonstrate that CNT incorporation significantly improves the hydrogel’s tensile strength and extensibility. Sensors integrated into clothing or attached to the skin undergo complex and repetitive deformations during daily activities or exercise, and Figure 2 visually confirms that this hydrogel can withstand such deformations without failure, autonomously restoring its original shape, thereby recovering its mechanical properties after deformation, and achieving consistent and reliable long-term performance. This supports the suitability of this CNT/CNP/PVA–borax hydrogel for manufacturing durable, reliable wearable pressure sensors capable of stable operation in dynamic environments.

#### 2.1.3. Adhesion of CNT/CNP/PVA–Borax Hydrogel

**Figure 3:**
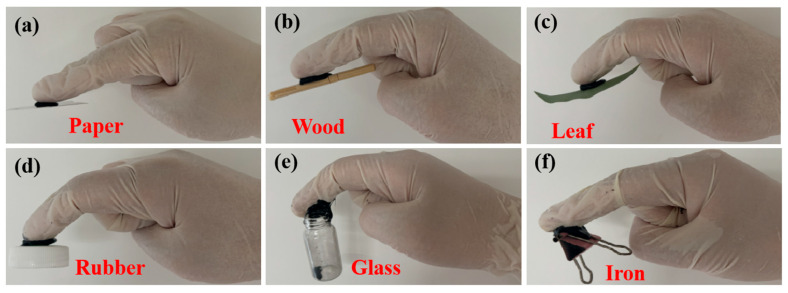
Photographs demonstrating the adhesion of the CNT/CNP/PVA–borax hydrogel to various materials: (**a**) paper, (**b**) leaf, (**c**) wood, (**d**) plastic, (**e**) small glass vial, and (**f**) iron clip.

Figure 3 shows the adhesion performance test results of the CNT/CNP/PVA–borax hydrogel. This hydrogel exhibits strong universal adhesion properties, capable of firmly adhering to a wide range of heterogeneous substrate surfaces, including natural organic materials (wood, leaves (Figure 3b,c)), cellulose-based materials (paper (Figure 3a)), polymers (plastic, Figure 3d), inorganic materials (glass, Figure 3e), and metals (iron clamp, Figure 3f). This cross-material adhesion capability stems from the synergistic interaction between the dynamic borate ester bonds and hydroxyl groups in the PVA–borax system. The former provides reversible covalent cross-linking at the interface, while the latter enhances non-specific adsorption through hydrogen bonding, thereby achieving a strong, peel-resistant bond on complex surfaces. This property not only ensures long-term stable wear of the device on human skin or clothing but also adapts to shear deformation during movement.

### 2.2. Sensor Performance of CNT/CNP/PVA–Borax Hydrogel

**Figure 4:**
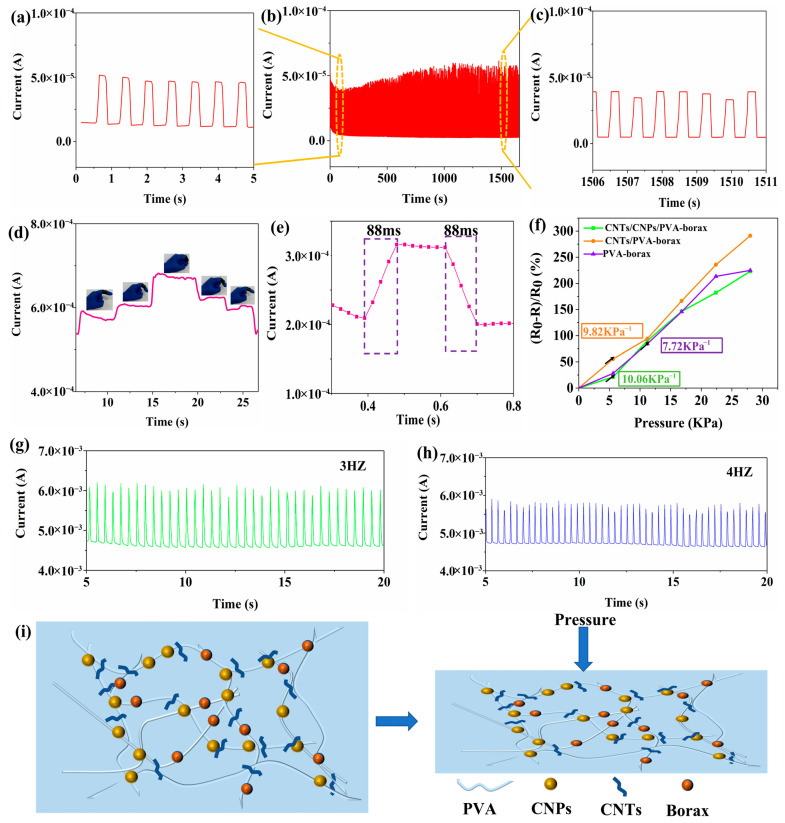
Sensor performance of the CNT/CNP/PVA–borax hydrogel: (**a**–**c**) Cyclic stability tests; (**d**) Relative resistance change in the sensor upon finger bending to 30°, 45°, and 90°; (**e**) Response and recovery time of the sensor; (**f**) Sensitivity of the sensor; (**g**) Stability test under dynamic bending cycles at 3 Hz; (**h**) Stability test under dynamic bending cycles at 4 Hz; (**i**) Piezoresistive working mechanisms.

Figure 4a–c show the sensing current response of the CNT/CNP/PVA–borax hydrogel under cyclic pressure of 2 Hz and 3200 fatigue tests. Figure 4a focuses on the initial 0–5 s of the cycle, clearly showing the periodic, instantaneous, and reversible changes in current with pressure loading/unloading. Each peak corresponds to pressure application, and each trough corresponds to pressure release. The waveforms are highly repetitive, indicating that the material exhibits excellent response sensitivity and instantaneous recovery capability at the microscopic time scale. Figure 4b depicts the macro-scale long-term trend, showing relatively stable changes in current. Figure 4c examines the current response during the final stage (1506–1511 s, near the 3200th cycle), whose waveform characteristics are highly similar to those of the initial cycle in Figure 4a. Although the peak current amplitude exhibits slight attenuation, it maintains stable periodicity and reversibility. Comprehensive analysis indicates that the core sensing functionality of this hydrogel sensor is well maintained even after undergoing up to 3200 fatigue tests. The excellent fatigue performance of the hydrogel demonstrated in the above test results is attributed to the self-healing capability conferred by the dynamic reversible cross-linking of the PVA–borax network, which effectively suppresses the irreversible accumulation of micro-damage, ensuring the structural integrity and functional stability of the conductive network under cyclic stress. This enables the hydrogel to exhibit outstanding fatigue resistance and long-term reliability in wearable sensor applications. The response performance of the CNT/CNP/PVA–borax hydrogel sensor when monitoring finger joint movements is shown in Figure 4d. This figure depicts the real-time curve of the sensor’s relative resistance changes during the process of the finger undergoing periodic bending (at angles of approximately 30°, 45°, and 90°) and subsequently straightening back to its initial position. It can be clearly observed that whenever the finger bends to a specific angle (30°, 45°, or 90°), the sensor’s relative resistance synchronously produces a significant peak response, and the amplitude of this peak is strictly positively correlated with the bending angle—the larger the bending angle, the higher the peak increase in resistance. When the finger completes the bending action and straightens back to its initial state, the relative resistance value of the sensor also rapidly and completely returns to the baseline level, demonstrating that this hydrogel sensor possesses excellent instantaneous responsiveness and deformation recovery capability. The resistance peaks generated by 45° and 90° bending exhibit noticeable differences in amplitude, enabling the hydrogel sensor to precisely distinguish and quantify different magnitudes of joint movement. This lays a reliable foundation for subsequent precise identification and monitoring of complex gesture or motion amplitude changes. When evaluated at a frequency of 2 Hz, the sensor has a fast response time of 88 ms (*n* ≥ 5 standard deviation 88.4 ± 0.6 ms) and a recovery time of 88 ms (*n* ≥ 5 standard deviation 88.2 ± 0.5 ms) (Figure 4e). In addition, the multiple resistance reduction mechanisms triggered by the structural changes in the conductive network of CNT/CNP/PVA–borax (10.06 kPa^−1^) were synergistically enhanced over the pressure range (0–28 kPa), resulting in better sensitivity than those of the CNPs/PVA–borax samples (9.82 Kpa^−1^) and PVA–borax samples (7.72 Kpa^−1^) (Figure 4f). The CNT/CNP/PVA–borax hydrogel sensor exhibits highly stable voltage potential output within the 3–4 Hz dynamic stress frequency range (Figure 4g,h). It demonstrates stable electrical signals under testing at different frequencies. In order to clearly explain the mechanism of the piezoresistive response of the CNT/CNP/PVA–borax sensors, the structure of the conductive network is schematically constructed in Figure 4i. Under pressure, the CNPs act as dot-bridging agents to enhance the connections between the CNTs and form a denser conductive pathway. The pressure brings the conductive fillers closer to each other, leading to an increase in the number of contacts and an increase in the tunnelling effect, which reduces the overall electrical resistance, demonstrating a typical point–wire synergistic enhancement mechanism. Table 1 presents a selection of recently reported hydrogel-based flexible sensors. The CNT/CNP/PVA–borax hydrogel pressure sensor, in particular, demonstrates excellent sensitivity and rapid response characteristics.

### 2.3. Self-Healing Properties of CNT/CNP/PVA–Borax Hydrogel

**Figure 5:**
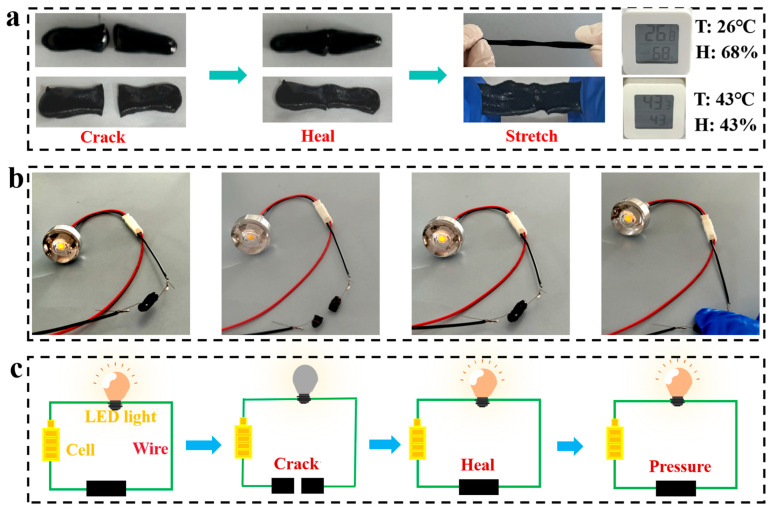
Characterization of the self-healing properties of CNT/CNP/PVA–borax hydrogel: (**a**) State changes during healing of cuts at room temperature with high humidity and at high temperature with low humidity, (**b**) Optical photos of an LED circuit based on the hydrogel at different healing stages, (**c**) Schematic diagram of circuit connectivity during the cutting–healing process.

Figure 5 visually demonstrates the synergistic recovery of mechanical properties and electrical conductivity during the self-healing process of CNT/CNP/PVA–borax hydrogel. Figure 5a shows the self-healing performance of the hydrogel under varying humidity and temperature conditions. The hydrogels were able to reconnect after being cut at 26.8 °C with 68% humidity and at 43.3 °C with 43% humidity. After the healing treatment, they could also withstand tensile stress, demonstrating their excellent self-healing capability under different environmental conditions. The healing efficiency of the hydrogel was 89.82%, which demonstrated the excellent self-healing ability of the hydrogel. The recovery of electrical properties was evaluated using a circuit connected to an LED (Figure 5b): the intact gel could illuminate the LED, attributed to its good conductivity; after cutting, the LED turned off; crucially, once the self-healing process was completed, the LED immediately recovered to nearly its initial brightness, confirming that the hydrogel can efficiently restore its initial conductivity after repair. The core mechanism of this synchronous repair stems from the highly reversible borate ester bonds formed by borax cross-linking in the PVA–borax dynamic network: after cutting, the exposed free hydroxyl groups at the fracture surface interact with borate ions, triggering a dynamic bond-breaking and re-forming reaction, driving the migration and interweaving of PVA molecular chains to achieve structural repair at the molecular level. Concurrently, the restoration of conductivity relies on a dual effect: on one hand, the reconstruction of the dynamic network provides a migration carrier for CNTs and CNPs nanofillers, enabling them to move with the matrix to the fracture site; on the other hand, the originally uniformly dispersed CNTs and CNPs are re-aligned at the newly formed interface through PVA adsorption, reconstituting the electronic transport pathways spanning the repaired interface (Figure 5b, lamp relighting). This unique synergistic mechanism endows the material with the ability to simultaneously restore mechanical integrity and conductive functionality at room temperature without external stimulation, aligning with the urgent demand for instant repair in flexible electronic devices. Figure 5c shows the simulated circuit of Figure 5b.

### 2.4. Applications of CNT/CNP/PVA–Borax Hydrogel Sensors

**Figure 6:**
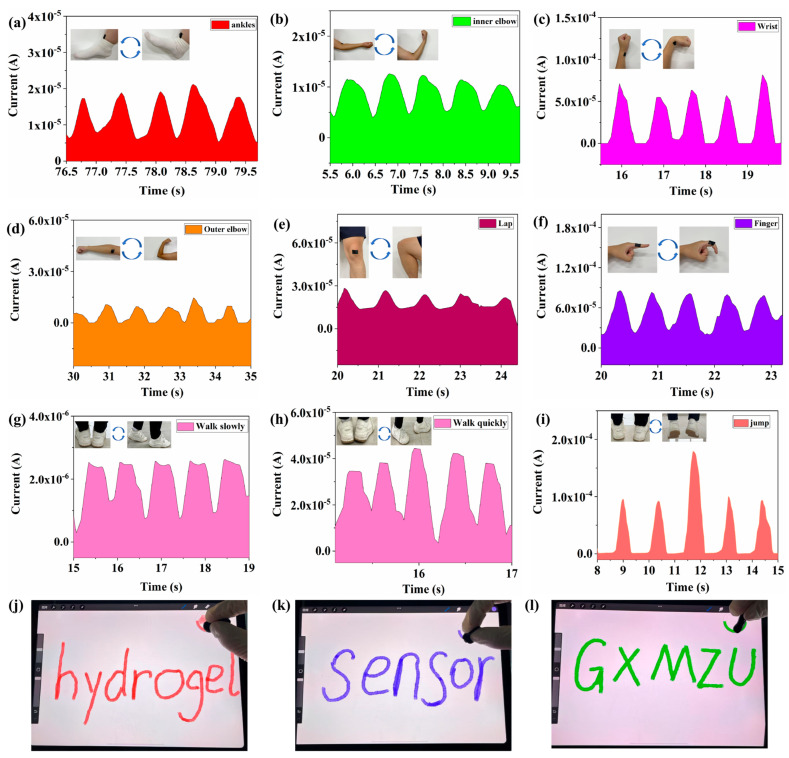
Motion monitoring and touchscreen writing using CNT/CNP/PVA–borax hydrogel sensor: (**a**) ankle flexion, (**b**) inner elbow flexion, (**c**) wrist flexion, (**d**) outer elbow flexion, (**e**) knee flexion, (**f**) finger flexion, (**g**) walking slowly, (**h**) walking quickly, (**i**) jumping, and (**j**–**l**) stylus made of the gel for touchpad interaction.

Due to its excellent conductivity, stretchability, sensing capability, and self-healing properties, the CNT/CNP/PVA–borax hydrogel was further developed into a flexible strain sensor for detecting human joint motion. This sensor can convert joint deformation into easily observable and analyzable electrical signals in real-time (Figure 6). As shown in Figure 6a, the hydrogel sensor was attached to the left ankle of a volunteer who performed rhythmic bending motions. The sensor’s output current signal correspondingly exhibited regular changes. Furthermore, when fixed onto the volunteer’s elbow (medial and lateral sides) and wrist (Figure 6b–d), the sensor responded rapidly and generated corresponding electrical signals during different arm bending movements. The height of the signal peaks clearly distinguished between different motion states. This sensor is equally suitable for detecting both large-scale and small-scale joint movements. For instance, during knee-bending (Figure 6e) and finger-bending (Figure 6f) tests, the sensor produced stable signals with distinct amplitudes, effectively reflecting the degree of joint movement. Benefiting from its exceptional plasticity, the CNT/CNP/PVA–borax hydrogel can be reshaped into various forms as needed. Figure 6g–i demonstrates the ability of the hydrogel sensor to capture the signal response under different gait patterns. As shown in Figure 6g, during slow walking, the sensor clearly detects distinct periodic signal peaks corresponding to each step. In Figure 6h, the signals become denser and more frequent under fast walking conditions, demonstrating the sensor’s ability to respond to fast motion. Furthermore, Figure 6i shows that the sensor maintains accurate signal detection even during jumping. These results indicate that the sensor is well suited for monitoring a wide range of gait patterns and is promising for health monitoring and motion analysis applications. A stylus fabricated on this basis successfully performed various gesture commands on a smartphone (Figure 6j) and drew complex patterns, including “hydrogel,” “sensor,” and “GXMZU,” on a touchpad (Figure 6k,l). The outstanding electrical performance demonstrated above, combined with its practical convenience, indicates that the CNT/CNP/PVA–borax hydrogel is a highly promising candidate material for applications in flexible electronic devices and electronic skin.

## 3. Conclusions

In summary, this study successfully developed a high-performance multifunctional wearable sensor based on a CNT/CNP/PVA–borax hydrogel. The synergistic integration of the PVA–borax network with carbon nanomaterials (CNTs/CNPs) endows the hydrogel with exceptional pressure-sensing capabilities. Comprehensive tests demonstrate that the hydrogel exhibits rapid and efficient self-healing, restoring structural integrity, and electrical conductivity after damage, due to its reversible borax ester bonds and stable conductive pathways. Simultaneously, it displays outstanding piezoresistive sensing performance, achieving an ultrafast response time of 88 ms and a recovery time of 88 ms, enabling real-time detection of subtle, rapid, and diverse physiological motions. Furthermore, the sensor demonstrates remarkable durability in fatigue resistance tests, maintaining stable sensing signals over 3200 continuous loading-unloading cycles, highlighting its superior mechanical properties and long-term reliability. The combination of rapid self-healing, ultrafast sensing response/recovery, high fatigue resistance, and excellent flexibility makes this CNT/CNP/PVA–borax hydrogel a highly promising multifunctional wearable sensor, capable of meeting the demands of advanced health monitoring and human–machine interaction applications.

## 4. Materials and Methods

### 4.1. Materials

Used polyvinyl alcohol (PVA) (hydrolysis degree: 87.0–89.0%) and borate buffer solution (0.005 mol/L, pH 9.3). The carbon nanotubes (CNTs) are multi-walled carbon nanotubes (wall thickness: 3–15 nm, length: 15–30 μm, purity > 97%), purchased from Shenzhen, China, Turing Evolution Technology Co., Ltd. The carbon nanospheres (CNPs) were prepared from cuttlefish ink (Qingdao, China, Xinjian Aquatic Products Co., Ltd.), with the preparation process including centrifugation drying and carbonization of the cuttlefish ink, among other steps. Deionized water (DI water) was prepared using a Milli-Q (Merck Group, Darmstadt, Germany)pure water system.

### 4.2. Preparation of CNPs

The cuttlefish ink sample was immersed in deionized water and soaked for 12 h. The resulting cuttlefish ink suspension was then centrifuged at 3000 r/min for 5 min to remove larger particles. On this basis, the suspension was further centrifuged at 9000 r/min for 10 min per cycle, repeated five times to ensure thorough purification. The obtained precipitate was dried at 60 °C for 24 h. The dried solid was ground in a mortar for 10 min to obtain a fine powder. This powder was then transferred into quartz boats and placed in a tube furnace. The temperature was increased at a rate of 5 °C/min up to 900 °C, and the sample was held at this temperature for 2 h under an argon atmosphere to synthesize CNPs.

### 4.3. Preparation of CNT/CNP/PVA–Borax Conductive Self-Healing Hydrogels

**Figure 7:**
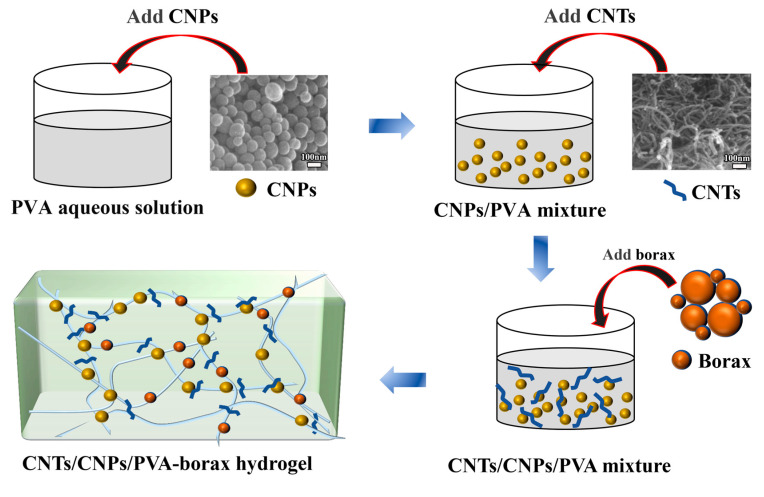
Preparation process of CNT/CNP/PVA–borax hydrogel.

A simplified diagram of the preparation process for the CNT/CNP/PVA–borax hydrogel is shown in Figure 7. The PVA (2 g) was dissolved in 20 mL of water at 90 °C for 1 h, followed by the sequential addition of predetermined amounts of 0.1 g CNPs and 0.1 g CNTs, with a CNT/CNP mass ratio of 1:1. After initial magnetic stirring for 10 min, the mixture was subjected to ultrasonication at a power of 250 W for 30 min to enhance dispersion. The resulting CNT/CNP-added PVA solution was then mixed with 2 mL of borax to form the CNT/CNP/PVA–borax hydrogel. This hydrogel was poured into a specially designed mold of a specific shape and size, frozen for 4 h, then removed and allowed to stand at room temperature for 4 h to obtain the final hydrogel macroblock.

### 4.4. Characterization

Observation of the microstructure of the carbon nanotubes and carbon nanospheres was conducted using a field emission scanning electron microscope (ZEISS Gemini Sigma 300, Zeiss, Germany). Fourier transform infrared spectroscopy (SHIMADZU, Tokyo, Japan) was used to detect the characteristic functional groups of the raw materials (PVA, CNTs, and CNPs), as well as the CNT/CNP/PVA–borax mixture. Cylindrical or rectangular hydrogels were cut into two or more pieces using a sharp blade. The newly cut surfaces were allowed to come into contact at room temperature without any external stimulation to observe the self-healing process and recovery of macroscopic integrity. The macroscopic hydrogel blocks were subjected to tensile, compressive, and torsional tests to study their mechanical properties.

Electrical signals from CNT/CNP/PVA–borax hydrogel were collected using a source meter (Keithley 2602 Cleveland, OH, USA). An Eidelberg HLD spiral machine (China, Yueqing City) stress tester was used to apply stable pressure to the hydrogel to detect its sensitivity. A cyclic stress-testing device (ShengDaMachinery China, Tianjin City) was used to test the fatigue performance of the hydrogel. NS-SourceMeters (2601B) software was used for monitoring the electrical signals of the hydrogel, recording the relative changes in current under compression strain of the hydrogel sensor, and evaluating key parameters such as sensitivity, response time, recovery time, stability, and repeatability under multiple cycles.

## Figures and Tables

**Table 1:** Performance comparison of various hydrogel sensors.

Raw Materials	Sensitivity (Kpa^−1^)	Sensing Range (KPa)	Response Time (ms)	Recovery Time (ms)	Ref
CNT/CNP/PVA -borax	10.06	0–28	88	88	This work
PVA/EMImAc/H_2_O	1.18	0.2–43	-	-	[36]
PVA/PANI	7.70	0–7.4	-	-	[37]
PVA/PAA/AG	0.121	0–31.83	-	-	[38]
PVA/CNTs/graphene	0.127	0–10	-	-	[39]
PDAX−PVA/PEG	0.57	0–30	210	330	[40]

## Data Availability

The original contributions presented in this study are included in the article. Further inquiries can be directed to the corresponding author.

## Abbreviations

The following abbreviations are used in this manuscript:

PVA	polyvinyl alcohol
CNTs	carbon nanotubes
CNPs	carbon nanospheres
one-dimensional	1D
zero-dimensional	0D
